# New Appliances and Things Medical

**Published:** 1901-02-16

**Authors:** 


### NEW APPLIANCES AND THINGS MEDICAL.

[We shall be glad to receive, at our Office, 28 & 29 Southampton Street, Strand, London, W.C., from the manufacturers, specimens of all new preparations and appliances which may be brought out from time to time.]

### MAGGI'S TONIC BOUILLON.

(LONDON AGENTS, COSENZA AND CO., 95 WIGMORE STREET, LONDON, W.)

THIS new departure in the preparation of a condensed meat extract will be thoroughly appreciated by invalids; for the bouillon, in addition to the usual stimulating and refreshing substances contained in similar preparations, possesses also an iron salt of valuable therapeutic properties. For persons suffering from anæmia or other complaints in which iron is indicated this preparation will be found a most agreeable medium for providing the required drug. In a previous number of this journal we had occasion to call attention to the artistic merits of Maggi's Consommé—no preparation with which we were acquainted being in the least degree comparable to it as regards delicacy of flavour. We can only repeat that the new tonic bouillon in no way falls short of the high standard attained by the original variety, and that no pleasanter clear soup could possibly be prepared even with the resources of a well-organised domestic kitchen. The Tonic Bouillon is supplied in small gelatine capsules packed in boxes of ten; by dissolving one capsule in a tea-cupful of boiling water the soup may be instantly prepared.

### HUNYADI JÁNOS.

(NATURAL APERIENT WATER. ANDREAS SAXLEHUES, TRAFALGAR BUILDINGS, CHARING CROSS, LONDON, W.C.)

AMONG the multitude of new mineral waters that are from time to time forced upon public notice old friends are apt to be forgotten. The medical mind has a limit, even where doses and therapeutic properties are concerned, and consequently it is easy to fall into certain grooves and habits in the prescribing of drugs or natural waters. Since Hunyadi János the parent of sulphated aperient waters—took up its position as the most popular of all remedial agents for the cure of constipation and plethoric conditions, many indifferent rivals have appeared upon the scene, and made bids for popular appreciation. Some of these waters are not natural waters at all, but are compounded from artificially manufactured salts and bottled for local consumption. The exact difference between a natural water and an artificial one of similar chemical composition has never been reduced to scientific expression; but that there is a difference is a matter of vulgar experience, and on chemical grounds it is probable that their difference is dependent on the arrangement of the component ions. Hunyadi János is one of the most reliable mineral waters on the market: it is practically of constant composition, and, thanks to the paternal care of the Government of the country whence it is exported, imitation or adulteration is almost impossible. The natural water is preserved at the wells from all sources of contamination, with the result that it reaches us practically free from organic matter. As repeated analyses have shown, the mineral contents are as follows: Sulphate of magnesia 160, sulphate of soda 159, sulphate of potash ·849, chloride of sodium 13, carbonate of sodium 8, per 10,000 parts of water, with traces of silicious earth and iron. It possesses, therefore, the ideal properties of a sulphated aperient water.

### MARVIS.

(THE PATENT FISH FOOD SYNDICATE, LIMITED, BERRY YARDS, GREENOCK, N.B.)

MARVIS is a new concentrated food prepared from fresh fish, the cod caught off the north coast of Scotland being used for the purpose. By a special process the nutritive portions of the fish are desiccated and reduced to a fine powder. Not only does Marvis contain a high percentage of proteid matter, but also certain salts which are of special value in many debilitated conditions. The powder may be used in several ways, but perhaps the most satisfactory method is to employ it as the nutritive basis of an ordinary soup or broth. If employed in this way it will afford to beef-tea or mutton broth the nutritive properties which are almost entirely absent unless some such addition is made. Of all proteid foods the flesh of fish is the least stimulating. For this reason it is particularly valued in cases of gouty dyspepsia. Under circumstances in which the combined action of a stimulant is desired Marvis should be given with the addition of such stimulating extractives as are present in soups made from butcher's meat.

### CRICINE POWDER AND CRICINE CREAM.

(THE CRICINE COMPANY, REGENT HOUSE, REGENT STREET, LONDON W.)

WE have examined samples of the above preparations, and find no objectionable or deleterious matter contained in either of them. The powder is finely divided, soft, and soothing to the skin. We recommend it for nursery or toilette use. Cricine cream is particularly emollient and suitable for delicate skins or those subject to roughness or chapping of the integument.

